# Death, social reform and the scrutiny of social welfare provision: the role of the contemporary inquest

**DOI:** 10.1080/09649069.2023.2281847

**Published:** 2023-11-26

**Authors:** Edward Kirton-Darling

**Affiliations:** University of Bristol Law School, University of Bristol, Bristol, UK

**Keywords:** Social welfare, social reform, death, inquest, coroner, article 2 ECHR, vulnerability, autonomy

## Abstract

Suggesting there is an emerging and important focus on social welfare in inquests into death, this article argues that there is value for both social welfare and inquest scholarship in examination of links between the two. Emphasising the process of investigation, it aims to introduce the inquest to social welfare scholars, and proposes an agenda for research. The discussion examines a range of inquests in which questions of social welfare (understood broadly) were examined, including inquests into the death of Jackie Maguire in a care home (see *R (Maguire) v. HM Senior Coroner for Blackpool & Fylde* [2023] UKSC 20), the death of Jodey Whiting after her welfare benefits were stopped (in a case brought by her mother, Joy Dove, see *Dove v. HM Assistant Coroner for Teesside and Hartlepool* [2023] EWCA Civ 289), the death of Awaab Ishak due to mould in his housing association home, and the death of Ella Kissi-Debra which suggested a link between traffic, air pollution and asthma, as well as other cases, including the inquest into the death of Molly Russell which focused on the role of social media.

## Introduction

One of the saddest indictments of this country I can think of is that the social reforms we need are written by coroners’

Tweet by Peter Apps, 10:43 on 16 November 2022

It was the conclusion of the inquest into the death of two year old Awaab Ishak that prompted Apps to reflect on social reform and the inquest. Deputy editor of the leading journal for housing professionals and author of a book on the Grenfell Tower fire (Apps 2022), Apps and his team at Inside Housing were reporting the coroner’s findings; that, despite pleas from his parents to deal with the problem, Awaab Ishak died as a result of mould in his home, and that – apart from blaming the lifestyle of his family – no action was taken to deal with the problem. The coroner concluded that placing responsibility on his family was entirely unfair; there was inadequate ventilation in the home and that ‘a more proactive response should have been taken to treat the mould which was present and to take steps to prevent its re-occurrence’ (Conclusion Ishak 2022).

To describe his death as a tragedy is to suggest it was a matter of fate, inevitable and inescapable. Instead Awaab Ishak died as a result of systemic factors and individual decisions, and while foreseeable, his death was contingent and preventable. In the contemporary context, it is the responsibility of the coroner and the inquest they oversee to highlight such wider matters of concern, to identify causes of death and to answer specific questions about the circumstances of a death, in a process which can trace its roots back to the twelfth century. Unsurprisingly, but contrary to some suggestions (Rozenberg 2004, vii), this is not a history of a static office, as the inquest has discreetly morphed through the centuries to embrace different functions; from the administration of taxes and criminal justice, to a local forum responsible for allaying rumour, suspicion and gossip about a sudden death, to a medico-legal role within the broader architecture of State provision for health, to responsibility for meeting human rights obligations and a focus on the needs of the bereaved. Often described as under-researched, there is scholarship on these aspects (see for example, Atkinson 1978, Hunnisett 1961, Scraton and Chadwick 1987, Prior 1989, Burney 2000, Easton 2020, Kirton-Darling 2022) but there has been no systematic analysis of what this article will suggest is an emerging but important focus of the contemporary inquest – the scrutiny of social welfare provision and questions of social reform.

Looking in two directions, to scholarship and practice in both social welfare and the inquest, this article aims to introduce the value of bringing these together; to set out some introductory reflections on what focusing on the inquest might add to social welfare scholarship and what an approach based in social welfare might add to the inquest. For those less familiar with the coroner’s court, the discussion therefore introduces and explores some of the key debates in the inquest jurisdiction, including making arguments for an understanding of the inquest which recognises the central role of bereaved kin in an investigation which includes meaningful scrutiny of the circumstances of death, and that it is the role of the inquest to unpick and reveal the need for social reform, not to make recommendations for change. It is a discussion which will focus on outlining the limitations and possibilities of the inquest in this context, and to propose an agenda for future research.

The analysis is in three substantive parts. It opens with discussion of the inquest, and explores links to social welfare to seek to substantiate the suggestion that these are issues which can be collectively identified as a focus of the contemporary inquest. It then moves to analysis of two different aspects of the coronial jurisdiction; the breadth (or ‘scope’) of a coronial investigation, and the official products of the inquest: the conclusions, and any ‘Report on action to prevent other deaths’ or Prevention of Future Death (PFD) report produced.

### The contemporary inquest

It is a common approach, when describing the inquest, to start with conclusions. The inquest cannot make findings of civil or (named) criminal liability and instead is required to answer four questions: who the person was, and how, when and where they died. Coroners (or a jury) cannot express an opinion on any other matter (Coroners and Justice Act 2009, hereafter CJA 2009, ss. 5 & 10). However, starting with these bald statutory statements produces a misleading impression. Liability may not be set out in the conclusion or directly alluded to in the public hearing, but contestation over it may be clearly evident, and it can be central to what evidence emerges (e.g. as a result of the rule against self-incrimination in R.22 of the Coroners (Inquests) Rules 2013 SI 2013/1616). Meanwhile emphasis on the four questions flattens the outcomes, presuming a box ticking uniformity which belies the varied products of the investigation. It also appears to suggest – incorrectly – that everything in the course of the inquiry is tied closely to narrow causative answers to the four questions, and so downplays the importance of additional duties to produce PFD Reports and requirements to ascertain other pieces of information as required by the Registration Acts (s.5(1)(c) CJA 2009), as well as the possibility in practice of allowing the bereaved to introduce materials about the life of the deceased or examine (potentially non-causative) aspects of the circumstances of death (Kirton-Darling 2022, pp. 153–4).

A more productive starting point is to approach the inquest system as a public mechanism for investigating the death of an individual, led by a coroner but with multiple potential participants who are likely to have a variety of different reasons for taking part. This is not to suggest the official conclusions of the investigation are not important, or to deny that the need to find conclusions will play a central role in decisions about which issues are explored in that investigation (as will be clear from the discussion of the breadth of the inquest below), but focusing on process reinforces the fact that it is a context in which ultimately fruitless avenues may be pursued and questions may be asked, in a public inquiry which ‘is almost bound to stretch wider than strictly required for the purposes of the verdict’ (*R v Inner West London Coroner, Ex Parte Dallaglio* [1994] 4 All ER 139 at 155b, Simon Brown LJ, and see Bingham MR at 164). This is essential for the inquest to fulfil its function ‘to seek out and record as many of the facts concerning the death as the public interest requires (*Sutovic)* it is to establish the “substantial truth” (*Hillsborough*)’. (*Dove v. HM Assistant Coroner for Teesside and Hartlepool* [2023] EWCA Civ 289 at 72, references as in original, hereafter *Dove*). In the words of Bingham MR (as he then was), it places a duty on the coroner ‘to ensure that the relevant facts are fully, fairly and fearlessly investigated’ (*R v HM Coroner for North Humberside, ex p Jamieson* [1995] QB 1 at 14, hereafter *Jamieson*) and to determine the breadth of the investigation (commonly known as the ‘scope’ of the inquest).

Decentering the official conclusion and instead emphasising the investigative process is important in two specific ways. Firstly, it highlights the other ways in which the inquest produces and promulgates knowledge which can have impacts beyond the coroner’s court, including the public airing of evidence and any PFD Report produced. At the same time, it draws attention to the contingent nature of the production of that knowledge, highlighting the need to focus on the unfolding of an investigation and the actors (including the bereaved) who play a role in determining whether and how information can emerge (or not), be explained (or not) and ‘social facts’ (Scott Bray 2010) are established (or not). As developed elsewhere in this issue by Khan, it necessarily focuses attention on the role of kin in particular, and the challenge each bereaved person faces of experiencing bereavement at the same time as being required to effectively engage with a process of formal investigation, including their ability to access and instruct legal representation and raise concerns linked to the death. Secondly, and closely linked to the first point, it places the inquest in a wider context, as potentially part of wider systems of accountability – political, legal, medical, administrative – and so emphasises links to those other mechanisms for revealing, explaining and requiring justification. It therefore directs attention to the ways in which inquests relate to other avenues of accountability and investigation, and the potential for undermining or supporting those other approaches. In the legal context, such investigations can precede and so potentially inform the inquest or limit the scope of its investigation, or might conclude afterwards. They might include criminal investigations and prosecutions or inquiries (including under the Inquiries Act 2005, see CJA 2009 Sch 1), civil claims, Safeguarding reports (see Coroners (Investigations) Regulations 2013, R.24), independent reviews like those undertaken by the Independent Case Examiner, investigations by ombudsmen or regulators like the Care Quality Commission, and internal investigations – all of which might be focused on the same circumstances as the inquest, or might be focused on subsequent but related situations. Placing the inquest in the context of these other inquiries is important to understand the ways in which – as explored further below – limits have been placed on the role of the inquest, but also frames it as one part of a complex network of systems involved with scrutinising the circumstances of an individual death. However, the inquest maintains important distinctions from other mechanisms, including the unique nature of the locally based coroner, the public nature of the inquiry they oversee, and the wide range of outcomes it can produce, including narrative conclusions, as well as – most importantly – the ability of the bereaved to be involved and to shape the direction of the investigation (on which see Kirton-Darling 2022).

Although cautious not to overstate the development of this area, it is a system which has undertaken some important investigations relating to social welfare provision and social policy. As discussed further below, inquests have examined the impact of removal of welfare support in individual cases and deaths relating to debt, while in the appellate courts, the two most significant inquest cases of 2023 relate to social welfare provision and what the inquest ought to investigate; *Dove* and *Maguire* (*R (Maguire) v. HM Senior Coroner for Blackpool & Fylde* [2023] UKSC 20). Education provision has been the focus of coronial investigations, for example, a three year wait for assessment of special educational needs in one case, which led to a coroner noting that

With finite resources, it is acknowledged that it may not be possible for all young people to be assessed in as timely a manner as required, but there must surely come a point whereby, notwithstanding those finite resources, the wait for assessment is taking too long. (Blackpool and Fylde PFD 2023

The regulation of employment has been raised in PFD Reports, as in the report linked to the death of Jennifer Davies, where the coroner noted concerns about the lack of regulation of working time for delivery van drivers driving long hours ‘in hugely populated areas where pedestrians are particularly at risk’ (PFD Davies 2022), while both employment practices and systemic questions about Ofsted seem likely to be scrutinised in relation to the death of headteacher Ruth Perry (Cumiskey 2023).

Meanwhile, inquests have examined actions of immigration enforcement officers, for example, Mustafa Dawood (also known as Mustafa Abdelkarim), whose death was found by a jury to have been contributed to by the actions of immigration enforcement officers (PFD Abdelkarim 2021) and the charity INQUEST track inquests which have examined deaths in immigration detention (INQUEST 2023b). Questions of community care (eg PFD Farran 2013) and care home provision have been examined, with 299 PFD Reports related to deaths in care home as of 8 August 2023 (Chief Coroner 2023). Finally, homelessness provision, and in particular, the provision of temporary and supported accommodation, has been the subject of two high profile inquests in the north east of England, touching on the deaths of Tina Robson and Ben Nelson-Roux (INQUEST 2022, 2023b 2023a).

These are areas of law traditionally understood as part of social welfare (see eg Adler 2022, pp. 2–3), but as well as providing a forum for the scrutiny of social welfare law, and a resource for research into social welfare law and policy, social welfare inquests might provide a productive lens to examine conceptualisations of the boundaries of social welfare law. As the conclusion notes, this is an area which requires further research, but the cases above might be understood initially as illustrating what Meers *et al*. (2023) describe as the ‘dustbin’ approach, in which social welfare law acts to bring together otherwise discrete areas of law. However, this would be to miss the ways in which the social welfare inquest focuses attention on links and cumulative impacts (Meers 2022). These might be between areas of law, both between areas of law traditionally within social welfare, and with areas not traditionally seen as part of social welfare as, for example, might be seen in the case of the inquest into the death of Ella Kissi-Debrah, which drew together consideration of health provision and urban development/planning (PFD PFD and Kissi-Debrah 2021, Barlow 2022). At the same time, the fact that the focus of the inquest is on the individual death means it is an investigation which can cut across regulatory systems or jurisdictional boundaries, to scrutinise fatal gaps or ‘frontiers’ in provision (Donnison 1976).

An example is the space between the NHS and local authority identified in the investigation into the death of Molly-Ann Sergeant, a 17 year old child in need who died on 16 October 2020. She had been detained under the Mental Health Act, and the inquest touching her death concluded on 7 December 2022 that

Social care failed to carry out appropriate requested assessments during Molly’s prolonged hospital admission and there was not a coherent co-ordinated approach to meeting Molly’s social aftercare needs. Molly’s right to aftercare services was recorded but the functions were not discharged as they should have been during her admission, and this contributed to her death (PFD Sergeant 2023).

Such gaps between services are common features of PFD Reports, as can be further illustrated by the PFD Report following the inquest touching the death of Reginald Cauthery: in which the relations between Care Act and fire risk assessments and the interface of public authorities and private telecare services were scrutinised (PFD Cauthery 2022). Inquest cases therefore provide a valuable resource for wider consideration of the structures of social welfare provision within the State and also ‘the relationship between the private and public sectors of the economy in relation to matters of social policy’ (Partington 1987, p. 422). Given the need to move beyond a focus on the State, as Meers et al argue Meers *et al*. (2023), analysis of social welfare inquests might enable this shift towards ‘the law of welfare governance’ (Meers *et al*. 2023, p. 205).

Although there is evidence in the historical record of (perhaps fleeting) concerns with social reform, including the description of some Victorian coroners as ‘The Magistrate of the Poor’ (Sim and Ward 1994), a lack of centralised publicly accessible inquest records and the local nature of the inquest system (Kirton-Darling 2022, pp. 26–29), with (currently) 82 separate coroner areas, does not make it straightforward to assess trends in the coronial jurisdiction. However, there is little evidence in scholarship or reported cases that social welfare provision was a particular focus of the inquest system in the twentieth century. In this context, further supporting evidence for a (potentially renewed) focus on social welfare might include the guidance from the Chief Coroner that ‘Broadly speaking, PFDs should be intended to improve public health, welfare and safety’ (Chief Coroner 2020, para 4), while it is notable that in both 2022 and 2023, the winner of the Legal Aid Newcomer award in the Legal Aid Lawyer of the Year awards was a barrister whose practice included a focus on inquests related to social welfare law: Ciara Bartlam (2022) and Christian Weaver (2023). This is particularly significant because of the longstanding close relationship between welfare law practice and social welfare scholarship, ‘evolv[ing] hand-in-hand’ as Meers et al put it (Meers *et al*. 2023, p. 202), and the fact that the inquest can be seen as a forum holding out the prospect of strategic legal action which cuts across jurisdictional boundaries (Kinghan 2021). However, obtaining such support, and opening up the ambit of the inquest, is extremely challenging in the context of high public funding thresholds for cases outside the classic Article 2 circumstances (discussed further below), making the role of the bereaved kin particularly significant (as discussed further by Khan 2023). In addition to those potentially insurmountable hurdles, other significant barriers which can undermine the possibility of detailed scrutiny in the social welfare inquest can include – the focus of the next section – the breadth of coronial discretion and individual decision making about the appropriateness of particular lines of investigation.

### The breadth of the investigation: revealing the substantial truth

Jodey Whiting was 42 years old in early 2017. As a result of problems which she faced with both her physical and mental health, she was in receipt of Employment and Support Allowance (ESA). On 6 February 2017, three weeks after she did not attend a face to face appointment, her ESA was stopped, which triggered the termination of other benefits she received. Reflecting on these decisions, Farbey J in the Divisional Court stated that she was

34…. bound to observe that the Department’s failures in this case … are shocking. ESA is not an unusual welfare benefit which might take staff away from familiar assessment tools and work methods: it is a mainstay of the social security system … In my judgment, the withdrawal of ESA should not have happened. (*Dove v. HM Assistant Coroner for Teesside and Hartlepool* [2021] EWHC 2511 (Admin))

Among other failures, there was no evidence that her mental health was taken into account and Jodey ought to have received a telephone call or, possibly, a ‘safeguarding visit’. A reinstatement decision at the end of March demonstrated that stopping her benefits was wrong, but this came too late, because on 21 February her mother found Jodey unresponsive on a sofa in her flat.

The inquest into her death was held on 24 May 2017, and, according to her mother Joy Dove, lasted 37 minutes (Dove 2022, p. 153). Ms Dove had attended with a photograph of her daughter as

I wanted the coroner and the witnesses to know that [Jodey] was more than a name and a statistic. I wanted them to see her face, to hear her story, and to know what we had lost (Dove 2022, p. 149).

However, she left ‘deflated’ (Dove 2022, p. 153). In her summing up, the coroner recorded that ‘Jodey had her ESA claim turned down in the weeks before her death and that her mother and sister believed that this caused her extra stress which was a contributing factor in her death’ (*Dove*, para 40) but stated during the hearing that ‘it was not the coroner’s position to question any decisions made by the Department and that was outside the remit of the Coroner’s Court’ (*Dove*, para 39). A single word conclusion of ‘suicide’ was recorded, with no other details about how, when and where she came by her death. Writing five years later, her mother described the impact of the hearing;

It had taken a little over half an hour to dispense with my daughter; her life and death. It felt like another stab in the back. I had built myself up to the inquest, hoping and believing that someone would be held accountable for Jodey’s death. Yet the hearing felt like a mere formality, as though her death was an inconvenience, a loose end, to be swept up and swept away (Dove 2022, p. 153).

It is not uncommon for those bereaved to (eg Ryan forthcoming) to feel frustration at the limitations of an inquest, including coronial decisions not to include particular issues within the investigation or the systemic inability to consider post-death failures. Driven by frustration with the DWP and a desire to tell her daughter’s story, Ms Dove started #JusticeforJodey, a campaign which included speaking at the 2019 Labour conference (Donnelly 2019). This campaigning brought her into contact with the charity INQUEST and Merry Varney, a solicitor who, amongst other cases, represented the family of Molly Russell, a 14 year old whose death was linked to the role of social media (PFD PFD and Russell 2022). Ms Dove applied for legal aid and then to the High Court seeking a new inquest, intending that ‘this time, if we were successful, the role of the DWP in her [daughter’s] death would hopefully be properly examined’ (Dove 2022, p. 179).

As Khan (2023) explains in more detail, the question for the courts was whether new evidence meant that a new inquest was ‘necessary or desirable in the interests of justice’ (S.13 Coroners Act 1988). The Court of Appeal held that it was, overturning a refusal by the Divisional Court, in a judgment which illustrates the interplay of expert evidence, coronial discretion, and the meaning of ‘scope’.

The first new piece of evidence obtained by the family of Jodey Whiting was a report by the Independent Case Examiner (ICE). Accepted by the Secretary of State, it identified failings including five missed opportunities to take Jodey’s needs properly into account. The question for the courts was what role the report should play. In the Divisional Court, the leading judgment by Farbey J and the short concurring decision by Warby LJ agreed on the point that an inquest was not necessary to seek further evidence about the failings. As Warby LJ noted, the report was

not a private or confidential document [and] I see no reason to believe that the ICE’s findings are incomplete or inadequate, or that a further coronial investigation is necessary or desirable to supplement them, or to provide further publicity, or for any other reason. (*Dove v. HM Assistant Coroner for Teesside and Hartlepool* [2021] EWHC 2511 (Admin) at para 100)

Existing in a space between publicly available official publication and private and confidential document, the nature of the report was not a significant issue for the Court of Appeal, who did not explicitly disagree on this point. Instead, they reframed the approach, holding that the issues raised were not irrelevant, and that it was a matter of discretion for an individual coroner to decide whether such a report ought to be admitted to an inquest; but

there is good reason why a coroner might wish to [admit it, to] … set the backdrop to the inquest accurately by establishing that Jodey should not have had her benefits stopped … But beyond acknowledging that fact, I doubt that the coroner would wish to investigate the Department’s conduct further; the specifics of individual errors and breaches of policies of the Department would appear to me to lie beyond the scope of any *Jamieson* inquest’. (para 64, CA)

*Jamieson* is a reference to the leading case for non-Article 2 inquests, and the approach of the court in *Dove* illustrates the potential subtlety of these decisions about the breadth of the investigation. Scope is graduated, so while particular issues may be central to the hearing or entirely excluded, others – such as in this case, the wrongful decision to stop benefits – might be explored in less depth, but there may be good reason to accurately set the context in the public record. However, it is a decision for the coroner, as the Court of Appeal reemphasised in 2018, noting that it

represents a coroner’s view about what is necessary, desirable and proportionate by way of investigation to enable the statutory functions to be discharged. These are not hard-edged questions. (*Coroner for the Birmingham Inquests (1974) v Julie Hambleton and Others v The Chief Constable of West Midlands Police* [2018] EWCA Civ 2081, at 48)

Scope is not a term found in the legislation, and is described by Matthews (2020, p. 133) as ‘having no special meaning … all that is generally meant is a list of the topics upon which the coroner, in the coroner’s discretion, will call relevant evidence so as to be able to answer the four statutory questions’. Exercising this discretion, and deciding what issues require evidence to be called, background or otherwise, might include, for example, asking the bereaved for information about the life of the deceased. A growing practice across the inquest jurisdiction, welcomed and endorsed by the Chief Coroner, this has come to be known as a ‘pen portrait’. Notably the Chief Coroner has described it as a practice which can help to answer the question of ‘who’ the deceased was, but has also (somewhat confusingly) stated it is ‘not a matter of evidence to be taken into account when deciding on the conclusion’. Either way, it is framed as something which can ‘humanise the process and give dignity to the bereaved’ (Coroner 2021b and regarding dignity, see, p.1, Kirton-Darling 2022, chapter 5), an objective which can be clearly contrasted with the way Joy Dove described her experiences of the inquest.

However, the coroner’s discretion, including on scope, is limited by public law principles, and in *Dove*, the second piece of new evidence, from a consultant psychiatrist which linked her state of mind to the withdrawal of her benefits, was ‘well within’ (*Dove*, para 65) the scope of an inquest. This enabled a departure from the reasoning of the Divisional Court, and required the Court of Appeal to consider the need for a new inquest. The leading judgment of Whipple LJ gave three reasons for ordering a new investigation; that it might be a case in which a PFD was appropriate (see below), that it was reasonable for the family to have concerns and wish to press for investigation of those concerns, and that it was in the public interest, as

[i]f Jodey’s death was connected with the abrupt cessation of benefits by the Department, the public has a legitimate interest in knowing that. After all, the Department deals with very many people who are vulnerable and dependent on benefits to survive, and the consequences of terminating benefit payments to such people should be examined in public, where it can be followed and reported on by others who might be interested in it. (*Dove*, para 72)

This emphasises the importance of wider public understanding, scrutiny and debate, in the context of widespread concerns about deaths linked to benefit withdrawal (Mills 2022) and 187 deaths prompting DWP internal reviews from July 2019-June 2023 (see Written Questions: UIN 27,621 and UIN 192,314, and see also Pring 2023, Mills and Pring 2023).

However, despite this wider public interest, it was not a case which engaged duties under Article 2 ECHR, including (potentially) greater scrutiny of systems and the wider context, or an expanded conclusion setting out the circumstances in which Jodey Whiting died. Such cases are described as *Middleton* inquests, following *R (Middleton) v West Somerset Coroner* [2004] UKHL 10, the case in which the requirements of Article 2 were incorporated into inquest law. Now given statutory authority by s.5(2) CJA 2009, the amalgam of human rights jurisprudence and domestic law means that where the Article 2 ‘enhanced procedural obligation’ arises, the conclusion can be stated in ‘judgmental’ terms, and while phrases suggestive of civil liability are impermissible – including negligence, breaches of duty, carelessness – conclusions can state, for example, that a particular action or failure to act contributed to the death (*Middleton*, para 20).

Contestation over when an inquest ought to reach an expanded conclusion including the circumstances in which the deceased came by their death has played out in inquest hearings and in higher courts since *Middleton*. This is because whether it will be a *Middleton* hearing or not has implications on the availability of legal aid (*R (Humberstone) v Legal Services Commission* [2010] EWCA Civ 1479), as well as potentially impacting on a civil claim (*Maguire*, para 30), as well as – potentially – on scope. The precise impact on the question of scope remains murky, and an explanation requires further examination of the approach of the courts in these circumstances.

Drawing on ‘young’ (*Rabone v Pennine Care NHS Trust* [2012] 2 AC 72, para 25) and ‘incremental’ (*Maguire*, para 34) Strasbourg jurisprudence, and in a series of cases which are not always easy to reconcile or understand, courts have analysed the ways in which procedural duties to investigate deaths relate to duties on the State to protect life, including in cases involving social welfare responsibilities. This includes the 2023 Supreme Court case relating to the inquest into the death of Jackie Maguire. She was a 52 year old woman with physical and mental disabilities who lived in a private care home paid for and supervised by Blackpool City Council and regulated by the Care Quality Commission. The chief concerns in the inquest included questions about whether paramedics, GPs and staff at the home failed to act appropriately to identify an infection in her stomach which led to her death, and in the course of the hearing, the coroner called evidence to explore a range of issues about the systems designed to keep residents of the home safe. The question for the Supreme Court was whether the coroner ought to have allowed the jury to return an expanded conclusion considering those circumstances, because – after hearing that evidence – the coroner concluded that the duty to do so did not arise as there was no arguable breach of the positive obligations within Article 2. The Supreme Court held unanimously that the coroner was right in their approach.

The leading judgment by Lord Sales and the concurring judgment of Lord Stephens examined two broad categories of substantive positive obligation: a duty to have systems to provide general protection for life (the ‘systems’ duty), and a duty to protect specific individuals from real and immediate risks (the ‘operational’ duty). They also identified and considered three procedural duties which arise under Article 2: (1) the obligation to take basic steps to confirm the cause of death, (2) the enhanced obligation (which will arise either automatically, eg in a non-natural causes death in prison, see *Maguire* para 16, or where there is an arguable breach of one of the substantive positive obligations), and (3) the redress obligation, which arises when there is ‘a possibility that the substantive obligations in article 2 have been breached’ (*Maguire*, para 19) but the enhanced obligation has been determined not to apply. ‘[T]ypically … applied’ in medical negligence cases (*Maguire*, para 19), this redress obligation can be met through the availability of a *Jamieson* non-expanded inquest plus a potential civil claim. Explaining the reasoning, Lord Stephens argued that this approach to cases involving arguable medical negligence ‘is not directed to carving out an exemption from article 2 in such cases, but reflects the lesser obligation of the state under that provision to exercise control and to give an account in that context’ (*Maguire*, para 148(3). It is, in essence, a policy choice by the courts, given the continuous real and immediate risks to life which feature in medical treatment and the potential burdens of having an enhanced duty for cases of ‘mere medical negligence’ (*Maguire*, para 46). From a systems-wide perspective, it means that the potential availability of a civil claim (irrespective of the challenges with bringing such claims) limits the operation of the inquest. In circumstances where death has occurred, and there have been errors which amount to medical negligence, the question is therefore whether there is something else in the circumstances, beyond those errors, which might amount to an arguable breach of the substantive positive obligations, and so give rise to an enhanced procedural obligation. In *Maguire*, the question for the Supreme Court was, how did this all operate in a social care context?

The argument for the family was that there was an arguable systemic breach as Jackie was under the care of the State which had direct responsibility for her welfare, and there was systemic dysfunction as she lacked capacity and there was no system for conveying her to hospital if she did not want to go. Furthermore, there was an arguable operational breach, based on the fact she was vulnerable, deprived of her liberty, and there was an assumption of responsibility for her.

These submissions were all rejected. The systems duty ‘operates at a high level, is relatively easily satisfied, and it will only be in rare cases that it will be found to have been breached’ (Maguire, para 145). Individual lapses will not breach this duty, and the court held that it was met by systems within the home and the supervisory role of the CQC. It ignores a traditional scholarly division between social welfare law and the law relating to health care (see Meers *et al*. 2023, p. 203), drawing the duty from healthcare into the care home setting and identifying the duty to maintain such systems in care homes is not higher than in a healthcare setting (*Maguire*, para 147).

Similarly, the threshold was not met by the actions of the paramedics or the GPs, as, while there were ‘lapses in individual performance’ (*Maguire*, para 184) by the GPs, in effect, the treatment overall did not constitute ‘a complete failure to provide basic medical assistance which was known to be required’ (*Maguire*, para 177). Likewise, there was no breach of the operational duty, as the care home staff took steps to access healthcare for Jackie (*Maguire*, para 204), while – in the view of Lord Sales – the paramedics acted reasonably in the circumstances (*Maguire*, para 208).

In analysis of all of these issues, classic questions for social welfare scholarship of the relationship of State responsibilities and individual vulnerabilities were relevant, and unpicking this analysis reveals important distinctions in the approach of the Court. For example, Lord Sales held that ‘The issue of assumption of responsibility [by the State] raises the question, assumption of responsibility for what?’ (*Maguire*, para 186). He concludes that assumption of responsibility for the care of an individual ‘does not involve an assumption of responsibility extending to taking responsibility for all aspects of their physical health’ (*Maguire*, para 190). The same approach was taken a few months earlier by the Court of Appeal in *Dove*, who held that

The fact that the Department is the agency responsible for administering the welfare benefits system does not of itself involve any assumption of responsibility to safeguard against the risks of suicide or self-harm by any of the many millions of persons with whom the Department has dealings. (*Dove*, para 91)

However, Lord Sales (or the Court of Appeal in *Dove*) did not adopt the same context-specific approach to vulnerability. It is instead treated as a fixed characteristic in much of the judgment; for example, ‘many people, young and old, share Jackie’s characteristics of being vulnerable and unable to care for themselves’ (*Maguire*, para 148(4)). The impact was that the opportunity to consider vulnerability as a relational term, to consider the question of what Jackie Maguire was vulnerable to, was not taken (see for comparison, the approach in *Hotak v London Borough of Southwark* [2015] UKSC 30, para 93). As the evidence shows, this was important because she was specifically, and particularly, vulnerable to medical mistreatment, due to various factors but importantly including her fear of and resistance to medical interventions as well as difficulties with having her needs effectively understood. This made her particularly vulnerable in circumstances such as those which led to her death, as contrasted for example with individuals who are physically frail or who lack capacity but are not fearful of medical intervention.

In contrast, Lord Stephens focused particular attention on her ‘personal circumstances to demonstrate her total dependence on others as to whether she should be treated at hospital’ (*Maguire*, para 214). His nuanced and compelling account outlines the specific ways in which Jackie’s vulnerability was important, identifying that she was vulnerable to mistreatment specifically because of her particular characteristics and needs. This analysis leads directly to his discussion of autonomy and dignity in an implicit critique of the approach of Lord Sales. Importantly, he found that while the care home staff and paramedics did not know there was an actual risk to her life, if they should have known, then there would be no question that considerations of her autonomy and personal dignity would outweigh the need to get her treated, and it would not have been an undue burden on the state to have sedated her and taken her to hospital (at a time when the expert evidence suggested that she had a 60–70% chance of surviving) (*Maguire*, para 229). He further notes that when she was eventually taken to hospital nothing had changed in terms of her capacity, but what had changed was actual knowledge of the nature of her condition (*Maguire*, para 232). However, critically,

after all the evidence was heard at the inquest, Ms Formby, for Jackie’s family, did not contend that there had been a breach of the *Osman* operational duty on the basis that the care home staff and the healthcare professionals ought to have known of the risk to Jackie’s life on 21 February 2017. (*Maguire*, para 230)

Furthermore, the coroner did not consider it. The implication is that, if it had been raised, the outcome of the Supreme Court decision might have been different, reinforcing the importance of the contingent emergence of evidence and decisions made in the course of the hearing, which in turn takes this discussion back to the scope of the hearing.

While the impact it has on the potential conclusions of the hearing is clear, the impact of the Article 2 enhanced procedural obligation on the scope of a particular inquest has not been conclusively determined. Obiter comments in the Court of Appeal suggest that there is ‘in practice little difference [between *Middleton* and *Jamieson* inquests] as far as inquisitorial scope is concerned’ (*R (Sreedharan) v. HM Coroner for Greater Manchester* [2013] EWCA Civ 181, para 18(vii)) and ‘The scope of the investigation and thus evidence called at the inquest is unlikely to be affected by the question whether the article 2 procedural obligation applies’ (*R (Maguire) v. HM Senior Coroner for Blackpool & Fylde and others* [2020] EWCA Civ 738, para 77). In contrast, Matthews submits that ‘there *is* a difference in scope’ (Matthews 2020, p. 140, emphasis in original), and that there are effectively three classes of cases: those where it definitely does or does not apply from the outset, and a middle category in which it is unclear and in which (he implies) it is safest to proceed on the basis of ‘cast[ing] the net wider rather than narrower’ (Matthews 2020, p. 141). It was not at issue in *Maguire* in the Supreme Court, but in obiter Lord Sales appears to support the position Matthews takes, noting that the ambit of the investigation must be kept under review throughout the course of the inquest, and information which emerges might result in the scope needing to be widened or narrowed as the case is heard (*Maguire*, para 32). However, the practice of a wider net as suggested by Matthews was specifically approved by Lord Stephens, who held that

Until an inquest is underway, and the real issues can be identified, there may be no proper way in which an assessment can be made as to whether there is an arguable breach of the state’s substantive positive obligations so as to engage the enhanced procedural obligation. Accordingly, the coroner will properly, as in this case, proceed on the basis that there is a need for an expanded verdict and then review the position at the end of the evidence. (*Maguire*, para 247)

The challenge of reflecting on the impact of these complex multilayered duties in real time during an inquest is significant, which may mean that, in practice, the suggested approach of Lord Stephens of reviewing at the end is easier to manage, and despite the adverse conclusions in *Maguire* and *Dove*, it will continue to be necessary to consider the application of Article 2 in similar cases. This is because while the threshold is high, the systems duty does include duty to have appropriate training and procedures in place (*Savage*, confirmed by *Maguire* at para 153), and the Court approved the ways in which coroner examined the specific systems for protection of life in detail. It is also necessary to consider whether, in any future case, there was a real and immediate risk, and whether there was an assumption of responsibility which was closely associated with that risk. It remains open whether, for example, a death linked to the termination of temporary accommodation which had been provided on the basis of vulnerability arising as a result of a risk of self harm or a specific health issue would meet the criteria of assumption of responsibility. Similarly, it might arise in a care home setting where staff do not act to seek medical assistance, or where – as suggested by Lord Stephens – the circumstances were such that those involved ought to have known of the risk. While, in Jodey Whiting’s case, the Court of Appeal found that there was no evidence ‘in any of her dealings with the Department in the weeks and days prior to her death’ (*Dove*, para 88) that her life was at real and immediate risk, it might have been a different outcome on different facts: had it been found that the DWP had such notice, or ought to have been aware.

This points to a key difference between *Dove* and *Maguire* – in the latter, the determination that Article 2 was arguably breached meant that the inquest had the benefit of lawyers representing the bereaved, able to assist the coroner in identifying and scrutinising evidence, while there was no such legal support for Joy Dove in the first inquest. Once it was obtained, it could pay for expert reports, enabling the issues to be properly explored. Joy Dove had to campaign and then litigate to have the issues re-examined, highlighting the importance of the role of the bereaved in what does and does not get investigated, what is part of the public debate, and on the function of seeking to prevent future deaths, as the decision illustrates;

Thirdly, if the findings the family seeks are made, it is at least possible that the coroner will wish to submit a PFD report to the Department. It is in the public interest that the coroner at least be given the opportunity to consider whether a PFD report is warranted, in light of the fact that Jodey’s benefits were cut off abruptly, in error, as we now know. If the coroner concluded that the error had contributed in any way, direct or indirect to Jodey’s death, that would be a serious matter to which the Department should be alerted, in order that remedial steps can be taken. Indeed, it may be that the coroner will wish to hear from the Department at the second inquest about any remedial steps which have already been taken in light of the ICE Report and as part of the coroner’s consideration of whether to make a PFD report. (para 72)

### Preventing future deaths and reaching conclusions

Examining the possibilities and limitations of the social welfare inquest inevitably involves engagement with the longstanding systemic objective of seeking to avoid future deaths. In 1980, the power that coroners and juries had been granted in previous Rules to add riders designed to prevent future deaths was abolished, and replaced with a power (for coroners alone) to make a report, where the coroner believed action might be taken to prevent the recurrence of similar fatalities. When the CJA 2009 was brought into force in 2013, this was transformed into a coronial duty to report to a person who may have the power to take action, with a duty on the recipient(s) to respond in writing (Para 7, Sch 5, CJA 2009). Importantly, the duty is not limited to highlighting issues which were causative in the immediate death under investigation or, unlike the previous rule, focused on similar circumstances, and further provision prescribing how the duty should be carried out is contained in regulations, including a timeframe for response of 56 days, and a duty on the recipient to give details of action they have taken or explanation of why no action is proposed (R. 28 & 29 Coroner (Investigations) Regulations 2013, SI 2013/1629).

Although it is commonly misdescribed as such, in England and Wales, it is not a responsibility to make recommendations to prevent such deaths (see Moore 2016 for other jurisdictions), and the absence of recommendations is one reason why calls for coroners to have the power to enforce a specific substantive outcome are flawed. Such a suggestion is also problematic because of the potential flaws and limitations of the inquest process, and because of a constitutional concern with the separation of powers, but the misconception that coroners do issue recommendations is understandable, as the line between reporting circumstances and making recommendations can be a thin one; illustrated by the PFD Report relating to the death of Kane Sparham-Price, sent to the Chief Executive of the Financial Conduct Authority. It recorded that

This young man had lived most of his short life in care homes and foster homes. He had numerous problems including those connected with his mental health. Having attained the age of 18 years he had, among other things, taken out pay-day loans with Wonga.com: On the day of his death Wonga had, within the terms of their agreement with him, taken out part payment of the debt from his bank account by using the debit card details they had been given. He was thus left with no money in his account and because part of the debt was outstanding he could not borrow any more. Later that day he was found hanging at his home address.

The Report goes on to set out the coroner’s concerns, stating;

Whilst I accept that the various pay-day lenders are legally entitled to ‘clear out’ someone’s bank account if money is owing to them, it struck me that there ought to be a statutory minimum amount which MUST be left in an account (say £10.00) to avoid absolute destitution; and as I understand you set and regulate the rules, you might look at this with a view to preventing future deaths (PFD PFD and Sparham-Price 2014). (formatting in original)

The FCA may be being invited to look at the issue, but where a potential solution is raised in which there ‘MUST’ be money left in an account, it is perhaps not surprising that the FCA understood it as a ‘recommendation’ (Financial Conduct Authority 2014). Their letter of response highlights a potential limitation of PFD Reports, as they describe a range of reasons why the suggestion would be unworkable (including privacy concerns), as well as setting out what action they had taken. It is unclear whether the FCA were called to give evidence to the inquest, but from the exchange of letters, it appears unlikely, and the letters therefore illustrate the ways in which the effectiveness of a PFD can be limited by the evidence which emerges in the inquest, which in turn can depend on a range of factors, including the capacity of the coroner to investigate, the evidence they are able to assemble and the role played by interested persons, including the bereaved. A contrast can be productively drawn with the inquest into the death of Luke Ashton (PFD Ashton 2023). It identified that he was suffering from a gambling disorder, and investigated the role of online operators, finding they did not make sufficient efforts to intervene when his problem worsened, with specific focus on issues with the algorithms and player protection tools utilised. In the PFD, the coroner remained

concerned that, as was apparent through the evidence of a senior employee witness during the course of the inquest, the operator Betfair appears to judge the extent of its responsibilities to gambling customers solely with regard to industry (regulatory) standards, rather than current good or best practice in order to prevent further harming problem gamblers, or those who, as a result of their changing practices and patterns are likely to become problem gamblers. (PFD Ashton 2023)

Unlike the concerns expressed following the death of Kane Sparham-Price, the careful interrogation of evidence at the hearing produced detailed and precise concerns. These were sent to a wider range of appropriate recipients, including the gambling operator but also the regulator, the Gambling Commission, the Secretary of State, the family of Luke Ashton and Gamble Aware, a charity concerned with gambling issues, with – it must be suggested – a much greater prospect of systemic changes which might save lives in future.

Academic analysis of PFDs (see inter alia Leary *et al*. 2021, Bremner *et al*. 2023, Zhang and Richards 2023), primarily focused on healthcare settings, highlights this potential value of PFD Reports, noting that they can ‘offer unparalleled insights into deaths due to patient safety errors in hospitals, by providing details not offered by other safety metrics’ (Bremner *et al*. 2023, p. 13). However, there is a clear need for more research on the ‘barriers and facilitators to writing and responding to PFDs’ (Bremner *et al*. 2023, p. 13). The research which does exist notes a range of issues including inconsistencies in the way PFDs are written, issues with a lack of response (or lack of publication of a response) and challenges in accessing the information on the Chief Coroner’s website. Further issues include a lack of a clear basis for the categorisation undertaken by the Chief Coroner’s office (Preventable Deaths Tracker 2023) and an absence of focused email alerts relating to specific topics.

More substantively, in relation to the possibilities and limitations of the social welfare inquest, the (potentially multiple) impacts, and timeframes for impact, is also under- researched and conceptualised, although illustrations of potential impact might include, for example, reflections on the collapse of Wonga in 2018, with the Economist noting that, four years on, ‘The death of Kane Sparham-Price came to symbolise all that was wrong with Britain’s “payday lenders”‘ (Economist 2018). Similarly, the subsequent impact of a report by a coroner might be seen in the ways in which the findings of the inquest into the fire at Lakanal House in 2013 (LB Southwark 2013 – ‘Verdicts and Coroner’s recommendations’ [sic]) have been identified as a critical part of the context for the fire at Grenfell Tower (Grenfell Tower Inquiry 2019, Apps 2022). However, these are instances of a report initially having little impact, and it is common, as Leary et al particularly note, to identify instances where coroners express concerns about needing to repeatedly raise the same concerns; in one example they note ‘The same coroner issued PFDs to one organisation in January 20 September 201414, November 20 December 201515 August 2016, January and March 2017 all regarding the delay of handover at an acute hospital due to resource issues’ (Leary *et al*. 2021, p. 17). The same issue can be seen with reports sent to the DWP from coroners arising from the deaths of Stephen Carre (Pring 2016) and Michael O’Sullivan (PFD PFD and O’Sullivan 2014) where both had been found to have died by suicide after flaws with ‘fitness to work’ tests.

The fire at Lakanal House is also an example of failures by the receipient to engage fully with the substance of the concerns laid out by the coroner (see Kirton-Darling 2018), an issue which is similarly evident in relation to the PFD reports sent following the death of Awaab Ishak. The coroner set out five matters of concern in her letter to the Secretaries of State for Housing and Health respectively (PFD Ishak 2022). Her fourth point of concern noted ‘a “policy” amongst the housing associations, in cases where a disrepair claim has been brought, of waiting for agreement from the claimant before rectifying any recognised disrepair’ (PFD Ishak 2022). In response, the letter from the Department for Levelling Up, Housing and Communities (DLUHC) notes that there is pre-existing pre-action guidance in place which states that landlords should not disengage from the repairs and flags a letter sent to all housing associations which stated they ‘must not hide behind legal process’ but notes that the Government ‘of course’ does not control the policies of Housing Associations (DLUHC 2023b).

However, the response then shifts, to note a letter which the Secretary of State wrote to legal advisors for tenants six weeks later, describing access to the law as a ‘vital right for all residents’ but noting that legal proceedings are adversarial, costly and time-consuming. The letter to legal professionals states

I am writing to ask for your help. I know how important the work you and your colleagues do is for people seeking justice. But I would like to emphasise the importance of directing social housing tenants with concerns about their housing to the Social Housing Ombudsman (DLUHC 2023a).

The response demonstrates the limitations of the PFD report process in which a matter of concern focused on the activities of housing associations can be transformed into an exhortation to lawyers to signpost inquiries elsewhere. In circumstances where claimants are legally entitled to pursue proceedings, it is not clear that it would be professionally appropriate for lawyers to direct tenants to the ombudsman. Furthermore, where the coroner specifically highlighted the issue with the approach of housing associations, it is troubling that the response suggests a shift of focus onto representatives for the tenants, but in the absence of any formal mechanism for reviewing PFDs, this is the end of the matter (at least as far as the inquest in this individual case is concerned).

Suggestions for improvement have included a requirement for follow up provision, in Parliament or elsewhere (see, e.g. Barlow 2022, pp. 179–180, INQUEST 2023c), while the Chief Coroner has produced guidance aimed at improvements within the current system, including a direction that

PFDs should not contain a detailed rehearsal of the facts of the death that has been the subject of investigation, or the history of the inquest. They are about learning. They should not contain personal information about the deceased, their family or others, that is unnecessary for the understanding of the learning points. An overview contained within a relatively short paragraph or two will usually be sufficient, followed by the specific points of concern (Chief Coroner 2020, para 5).

The approach echoes scholarly calls for consistency in reports, and it must be noted that the variety of approaches in PFD Reports published demonstrates that this emphasis on brevity is not necessarily being followed. However, there may be good reason for not following this approach in an individual case, as can be illustrated in the comments of one coroner interviewed by Kirton-Darling;

Coroner: I have always had this tendency to write long reports, but always with good purpose and always to explain why, because unless you tell a story people won’t understand why you are making your concerns.

Ed: Do you think it elicits a better response?

Coroner: In practice I think it does, because it is also unarguable, if you have got some vague nonsense, you get vague nonsense back, if you have told the story, it also means that the family know when I have taken so much care and time, that (1) the story has been told and (2) if the responses are inadequate then the families can then challenge and say ‘you haven’t responded, you haven’t adequately dealt with it. (Kirton-Darling 2022, p.16)

The response directs attention to the potential audiences for a PFD report, and highlights that while formal enforcement mechanisms may be limited, reports can be followed up, depending in part on the effectiveness of the story told by the PFD, its accessibility and an awareness of potential audiences and avenues for change. For example, it is notable, and welcome, that the coroner in the case of Luke Ashton sent a copy of the report to Gamble Aware. Where scholarship, particularly with a medical lens, has tended to focus on the potential impact on the recipient of a PFD, more attention might be productively focused on other potential audiences and their role, with a conception of pathways to change which are not limited to technical, internal tweaks to systems.

This emphasis on reflecting on the audience and accessibility also applies to the official conclusions of the inquest, and, it is suggested, can be seen as part of the reason for a growth in narrative conclusions in comparison to the traditional short-form. This ‘conclusion’ is recorded in Box 4 of the prescribed form used to record the outcome of the inquest (see Coroner (Inquests) Rules 2013, SI 2013/1616, Schedule, Form 2, which includes potential short form conclusions in the notes), and is one of the ‘determinations’ (s.10 CJA 2009) to be made at the end of the inquest. It must follow from the findings of fact and the answer in Box 3 of the form of how, when and where the person came by their death. As an example, the Record of Inquest in relation to the death of Luke Ashton is reproduced below:

**Figure:**
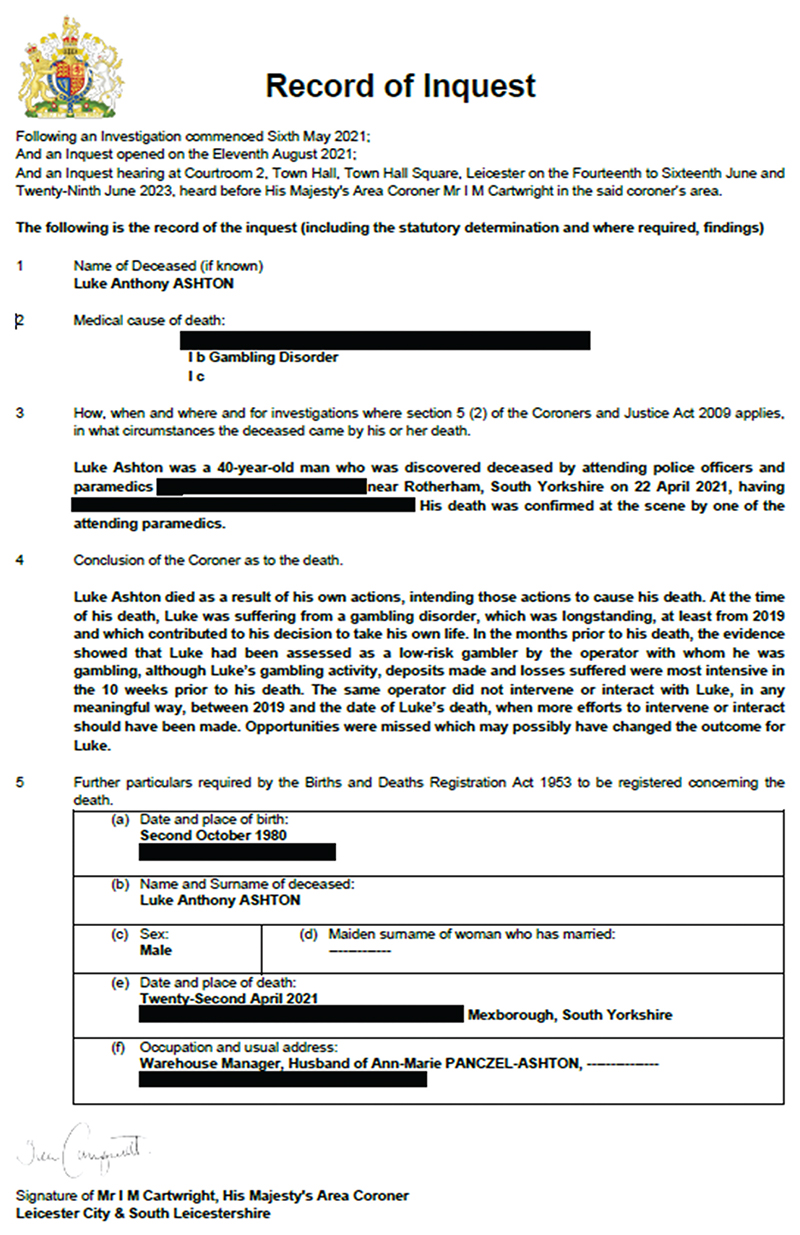


Record of Inquest, reproduced with grateful thanks to the family of Luke Ashton

The subject of statisticians’ concerns because of the additional challenges with coding and the production of death statistics (Hill and Cook 2011), narrative conclusions now make up around a quarter of all inquest conclusions (Ministry of Justice 2022). It is a welcome development as a narrative allows for a more nuanced explanation of death, including, where there is more than one cause of death (where they will be ‘often’ required, *R (Longfield Care Homes Ltd) v HM Coroner for Blackburn* [2004] EWHC 2467 (Admin), para 31), and where a single word ‘short form’ conclusion plus a sentence explaining how the person died does not ‘record as many of the facts concerning the death as the public interest requires’ (*R. v. HM Coroner for South London, ex p. Thompson* (1982) 126 S.J. 625, as quoted in Coroner (2021a). The development is particularly associated with Article 2, but narratives pre-date the Human Rights Act (see *Jamieson*, 22B) and are not limited to cases which engage Article 2. It was not engaged in Luke Ashton’s case for example, and similarly, in *Dove*, the court suggested that the expert evidence

would undoubtedly assist the coroner in deciding whether to enter a narrative conclusion in addition to, or an alternative formulation of, that conclusion, to reflect the extreme anxiety and distress that Jodey might have been suffering in the moments before she took her own life. (*Dove*, para 65)

The Court went on to note that the conclusion might include reference to the contribution made by the sudden withdrawal of benefits,

within the conclusions part of the record of inquest (by including it at box 3 or as part of a narrative conclusion in addition to or substitution for the short-form conclusion of suicide in box 4). Mr Hyam suggested, by way of example, a narrative conclusion along the lines of: ‘the deceased took her own life as a result of a deterioration in her mental state exacerbated by the abrupt cessation of her ESA on 7 February 2017 by the Department’; that is a brief, neutral, factual statement which I accept would be open to a coroner to adopt

For Joy Dove, such a conclusion might importantly recognise the role played by the DWP in her daughter’s death. The excerpt also emphasises the variety in the ways the inquest might produce explanations for death in the different parts of the form the coroner (or jury) is required to complete. Even if there is a short-form conclusion such as ‘suicide’ in Box 4, more explanation might be provided in Box 3, and further discretion is granted to the coroner who might choose a short form conclusion other than those suggested in the Rules (although the Chief Coroner suggests that to do so ‘will usually be unwise’, Coroner 2021a, para 16). Furthermore, it was held to be not necessarily unlawful where a jury chose to add handwritten notes setting out particular facts on a conclusion, and so drawing attention to particular aspects of the evidence (*R (Hamilton-Jackson) v HM Assistant Coroner for Mid Kent and Medway* [2016] EWHC 1796, para 66).

The potential for public explanation and subsequent impact in narrative conclusions has been explored by Baker (2016, 2019), who uses narrative conclusions to analyse deaths after police contact, noting that, in comparison to short form outcomes, they produce ‘a significant amount of detailed findings …, which in turn means that we know more about the circumstances involved …, and can thus consider potential interventions’ (Baker 2019, p. 62). One problem is the accessibility of such conclusions as they are not centrally collated or made public. This makes the role of the bereaved, interested organisations and the media critical in their wider promulgation; for example, the conclusions of the inquest touching the death of Awaab Ishak were only available online via a popular housing law blog (Conclusion Ishak 2022) or from Inside Housing (now behind a paywall).

More broadly, there is a significant research gap in relation to narrative outcomes from inquests (incorporating both official narrative conclusions and ‘Box 3’ narrative descriptions provided in addition to short form conclusions). Such research might, for example, productively place these conclusions in the context of research on narratives and impact more widely. For example, Bates *et al*. (2023) examine what kinds of evidence best persuades policy makers in an urban development context, and identified narratives as the most effective means. In particular, they highlight the accessibility and persuasiveness of relatable and contextualised but logically and rationally developed real life stories, and note that it is not inappropriate for emotion to be included in such accounts. The research included 132 indepth interviews with 123 actors as part of the TRUUD project with a variety of stake-holders with backgrounds in urban development including planning consultants and planning officers. Details of the methods can be found in Bates *et al*. (2023), but it is notable for the purposes of this article that in several of these interviews (unpublished elsewhere), interviewees, including planning officers and consultants, mentioned the case of Ella Kissi-Debrah and the links identified by the coroner between her death and air pollution, noting that there could be long-term consequences and future similar cases;

that case in London of the child who died of an asthmatic attack and how the mother’s taking it through the courts and I think that’s a project that’s going – that’s a legal event that’s going to have a long-term change I think.

we’re probably still getting to understand the full ramifications of the decision of – that young girl that died in London, which was down to air quality, I think that’s probably still working its way through and whether particular groups start to challenge things more.

the case was made at the coroner’s that basically that young girl in the case was killed, basically by the quality of the air in her local area, which was fundamentally driven by air pollution, and primarily air pollution from road transport. I suspect it won’t be long before we have a similar circumstance in ((city)), because we have exactly the same, if not, worse, congestion, air quality and severance issues that exist in that particular part of London.

The extent to which these respondents are correct and the inquest conclusions will prompt change is unclear, with ongoing contestation over air pollution and traffic management schemes, but these are responses which frame the inquest as part of wider systems of social health and welfare, reinforcing the potential significance (and limitations) of the inquest in the social welfare context and the need for further research in this area.

## Conclusion

It is important to be clear about the limitations of the inquest, and the ways in which the operation of the law, the exercise of discretion or the problems discussed above with the outcomes can produce an inquiry which fails to meaningfully respond to a death, including where social welfare responsibilities are somehow engaged. As Khan (2023) explores, it is also a place in which the bereaved kin can find it hard to be heard. At the same time, examining the inquest as a site of social welfare law can be of value for scholars and practitioners of both social welfare and inquest law.

For those engaged with social welfare, focusing on social welfare inquests suggests that even the broad approach adopted by Adler (2022), including all the major areas of non-economic law, is not broad enough to capture social welfare in the inquest, which might coherently, for example, include areas such as planning (see, although not focused on the inquest, Hubber 2023, Ogilvie-Harris 2023). In contrast, conceptualising social welfare instead through Wickenden’s ‘common denominator risk’ approach as developed by Hupe and Hill (see Meers *et al*. 2023, p. 201), could frame the inquest as a key site for considering the ways in which social risks arise from the regulation and use of social media or online gambling, and for consideration of the means to protect against such risks. Using the inquest as such a lens suggests the need to look at governance, at links, gaps, frontiers and cumulative effects, and also the need to think about people and their stories within systems. It also highlights the role of public debate and scrutiny, and the role (and limitations) of the inquest in prompting or informing such debates, including the role of the media (see Binns and Arnold 2020) and the impact of the presence – or importantly, the absence – of bereaved individuals, with lawyers or without.

From the perspective of inquest scholarship, a focus on social welfare prompts questions about how longstanding lines of scholarship on state misuse of power and a lack of accountability on one hand and about accuracy of conclusions and the role of medico-legal expertise on the other play out in the context of social welfare responsibilities, as well as questions about how this relates to developing understandings of what might constitute a health issue justifying state action (Black *et al*. 2023). It suggests a need to attend closely to the as yet unexamined relationship between the local authority and the inquest system, and – as highlighted above – it suggests there may be value in drawing on social welfare socio-legal scholarship, including for example, scholarship on vulnerability or regulation and processes and systems of administration and accountability, described by Adler as adopting

‘a ‘bottom-up’ approach that focuses on the myriad of first instance decisions, most of which are never subject to appeal, rather than a ‘top-down ‘ approach that focuses on a small number of leading cases (Adler 2006, p. 635).

The suggestion that this is a growing focus of the inquest needs further research, as does the question of the potential impacts of the inquest on social welfare law, in a context in which inquest conclusions have had direct impacts on legislative reform (for example, in the cases of Awaab Ishak and Molly Russell). Such research might, for example, draw on the potential for ‘communicative generalisations’ (Cornish 2020), and would need to consider the role of narratives and audiences, and the importance of telling stories not simply formally stating outcomes, in reflections about the ways in which the process of investigating an individual death might eventually produce ‘the social reforms we need’.
